# A Case of Porcelain Gallbladder Showing High Levels of CA19-9

**DOI:** 10.7759/cureus.72583

**Published:** 2024-10-28

**Authors:** Hideo Kidogawa, Kazuhiro Ootubo, Junya Noguchi, Takatomo Yamayoshi, Kohji Okamoto

**Affiliations:** 1 Department of Surgery, Kitakyushu City Yahata Hospital, Kitakyushu, JPN

**Keywords:** chronic cholecystitis, common bile duct stone, elevated ca19-9, laparoscopic surgery, porcelain gallbladder

## Abstract

Porcelain gallbladder is a rare condition characterized by an extensive calcification of the gallbladder wall. It is associated with an increased risk of gallbladder cancer and elevated levels of tumor marker CA19-9, which is typically seen in pancreatic and biliary cancers. We present a case of a 60-year-old woman who presented with upper abdominal pain and nausea. Imaging revealed both a porcelain gallbladder and common bile duct stones. Initially, the patient underwent endoscopic retrograde cholangiopancreatography (ERCP) for the removal of the common bile duct stones. Notably, following the removal of the stones, the patient's CA19-9 levels were significantly elevated, reaching 6076 U/mL. Subsequently, she underwent a laparoscopic cholecystectomy.

Histopathological examination and immunostaining for CA19-9 were performed on the resected specimen. Histopathology revealed marked fibrosis, calcification, and xanthogranulomatous inflammation of the gallbladder wall without evidence of malignancy. Immunostaining showed strong CA19-9 positivity in the inflamed gallbladder mucosal epithelium. Postoperatively, the patient's CA19-9 levels returned to normal (17 U/mL).

This case highlights that a benign porcelain gallbladder can present with abnormally high CA19-9 levels, potentially mimicking malignancy. Reports of CA19-9 elevation in porcelain gallbladder are limited, and in most cases, significant CA19-9 elevation has been associated with malignancy. This case also demonstrates that benign conditions such as biliary obstruction, chronic inflammation, and xanthogranulomatous cholecystitis can cause significant elevations in CA19-9 levels. Therefore, careful differentiation and comprehensive evaluation are crucial for accurate diagnosis and appropriate management in such cases.

## Introduction

Porcelain gallbladder is a condition in which the gallbladder wall undergoes extensive calcification, giving it an appearance and hardness similar to porcelain. The incidence is rare, ranging from 0.06% to 0.8% of cholecystectomy cases [1,2], and it is more common in women, particularly in their 60s [3]. Porcelain gallbladder has been associated with an increased risk of gallbladder cancer, with reported malignancy rates ranging from 12% to 33% [1,4]. This case involves a rare instance of a benign porcelain gallbladder presenting with considerably high levels of CA19-9, a tumor marker typically elevated in pancreatic and biliary cancers, although it is also known to be elevated in various benign conditions. We report a case of porcelain gallbladder with markedly high CA19-9 levels, in spite of being benign, as confirmed by histopathology performed postoperatively.

## Case presentation

A 60-year-old woman was referred to our hospital with a 13-day history of upper abdominal pain and nausea. Her past medical history included only hypertension, for which she was taking antihypertensive medication. She was 162 cm tall and weighed 87 kg, with a BMI of 33.1. Her temperature was 36.5℃, blood pressure 115/65 mmHg, and pulse rate 80 beats per minute (bpm). The abdomen was flat and soft, with a negative Murphy's sign and no muscle guarding. She reported discomfort in the right hypochondrium.

**Figure 1:**
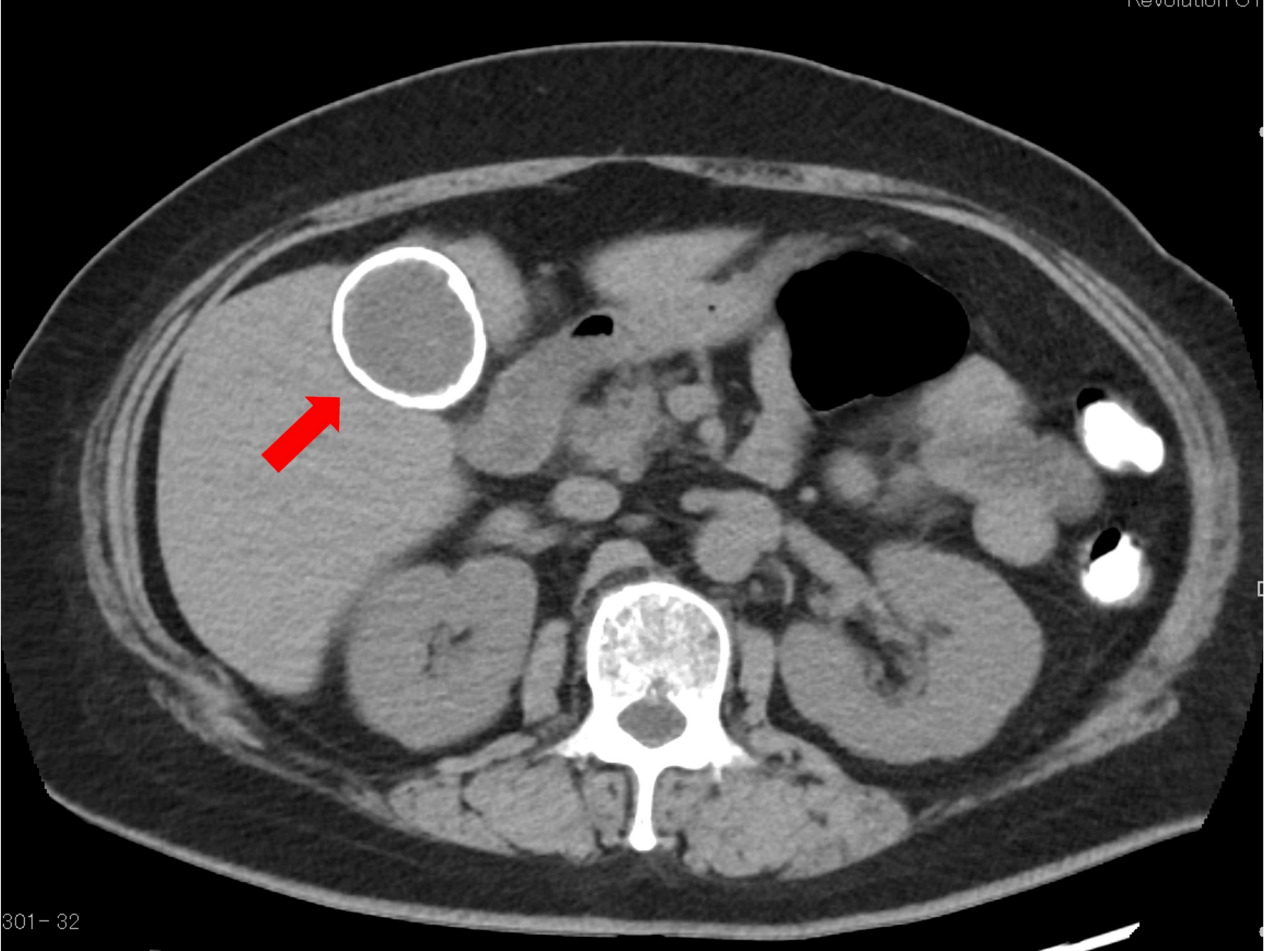
Axial view of the abdominal CT scan The axial view of the abdominal CT scan demonstrated calcification consistent with the gallbladder wall, forming a ringlike pattern (red arrow)

**Figure 2:**
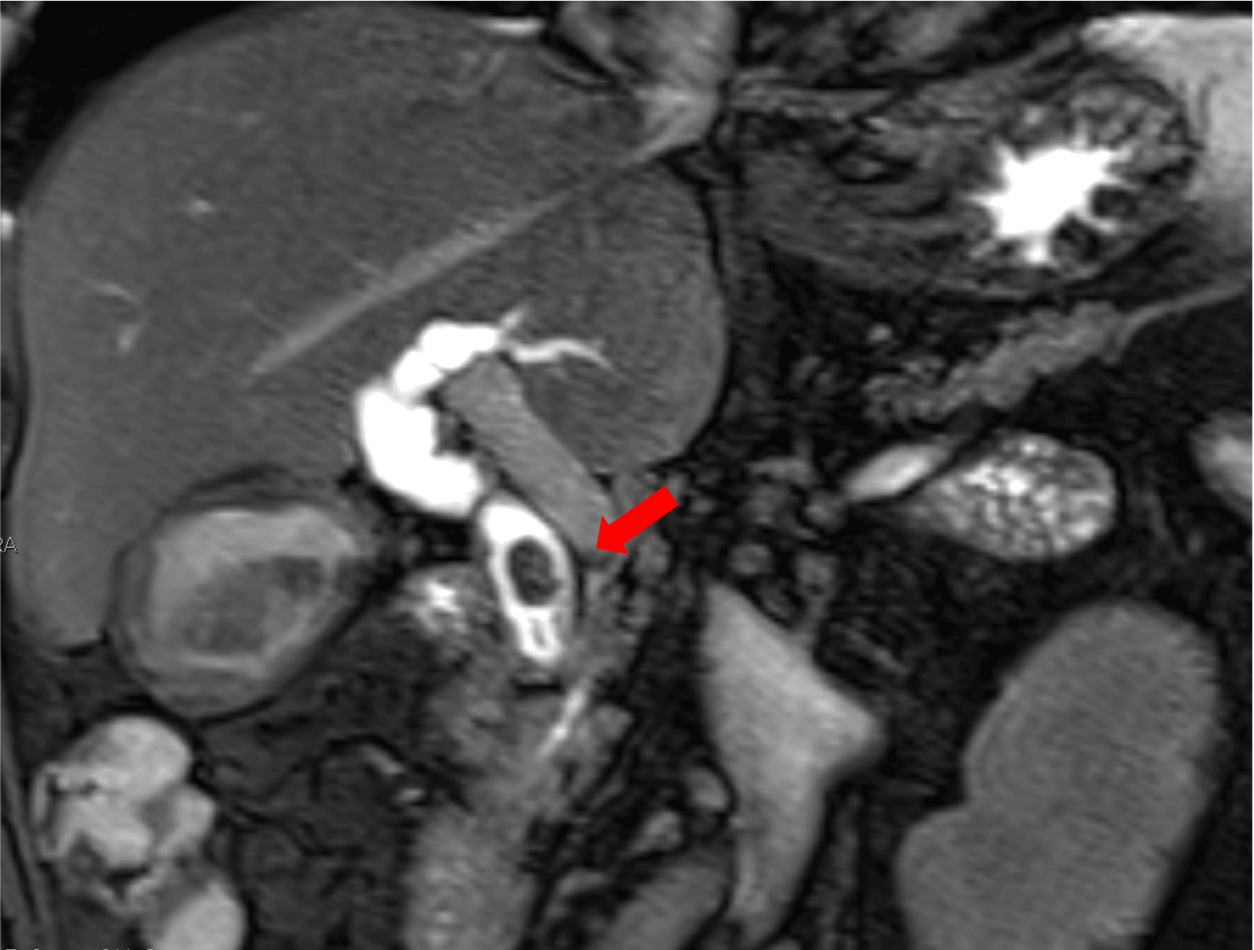
Coronal MRCP image Coronal MRCP image revealed multiple stones in the common bile duct (red arrow) MRCP: magnetic resonance cholangiopancreatography

Laboratory tests at admission revealed a white blood cell count of 9300/μL; CRP of 7.6 mg/dL, indicating inflammation; and elevated liver enzymes: total bilirubin (T-bil), 3.9 mg/dL; direct bilirubin (D-bil), 3.1 mg/dL; aspartate aminotransferase (AST), 32 IU/L; alanine aminotransferase (ALT), 53 IU/L; gamma-glutamyl transpeptidase (γ-GTP), 459 U/L; and alkaline phosphatase (ALP), 437 U/L. Abdominal plain radiography showed an eggshell-like calcification in the right upper abdomen. A plain abdominal CT scan demonstrated calcification consistent with the gallbladder wall, forming a ringlike pattern (Figure 1). Magnetic resonance cholangiopancreatography (MRCP) revealed multiple stones in the common bile duct (Figure 2).

Clinical course

**Figure 3:**
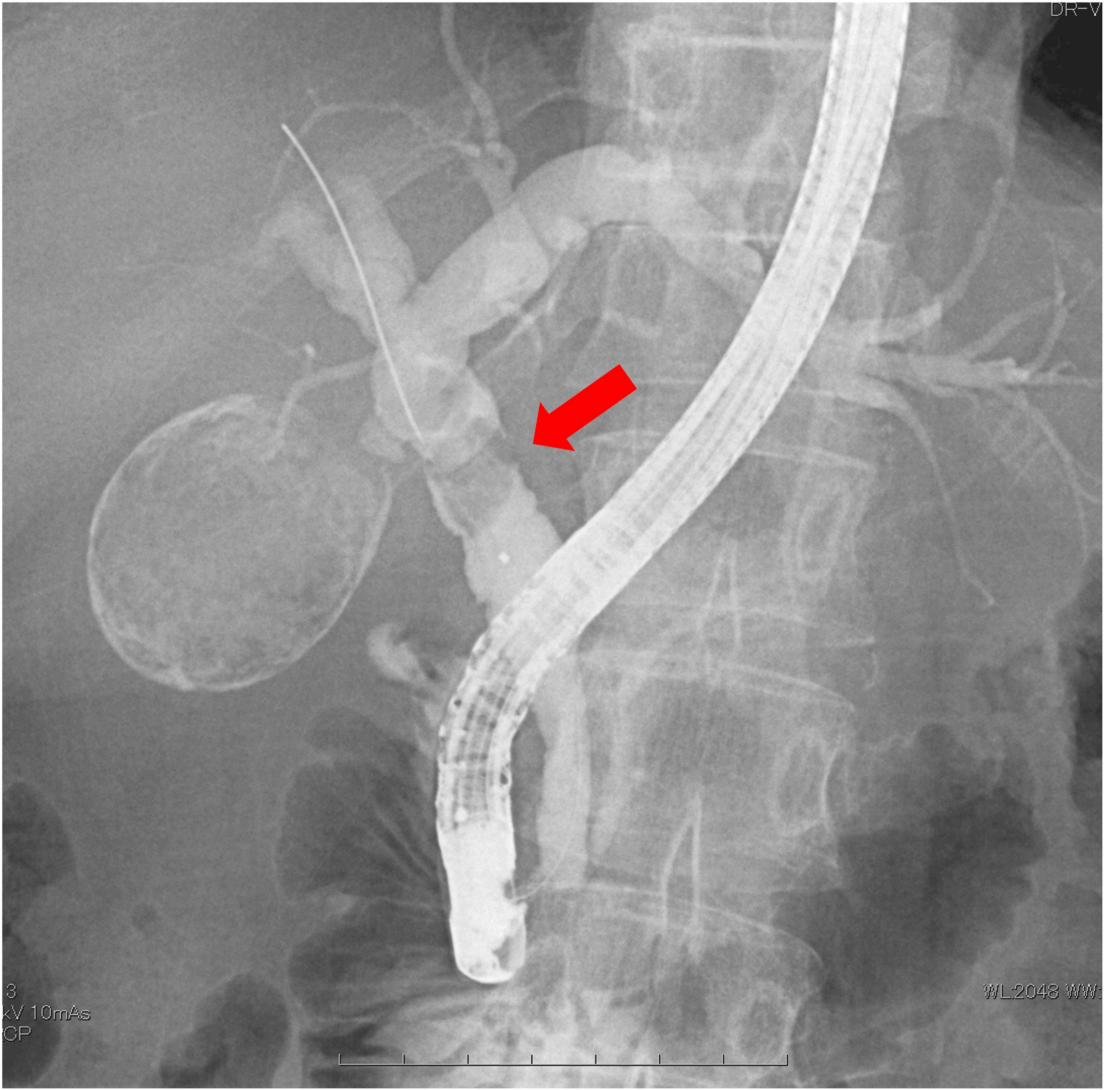
ERCP image showing common bile duct stones ERCP was performed, revealing two 10 mm stones in the common bile duct. EST was followed by lithotripsy and stone removal. Four days later, a second stone removal procedure was performed, successfully clearing the common bile duct stones ERCP, endoscopic retrograde cholangiopancreatography; EST, endoscopic sphincterotomy

On the day following admission, endoscopic retrograde cholangiopancreatography (ERCP) was performed, revealing two 10 mm stones in the common bile duct. Endoscopic sphincterotomy (EST) was followed by lithotripsy and stone removal (Figure 3). The CA19-9 level measured after ERCP was 3820 U/mL. Four days later, a second stone removal procedure was performed, successfully clearing the common bile duct stones. Despite further investigation, including upper gastrointestinal endoscopy, lower gastrointestinal endoscopy, and contrast-enhanced MRI, no evidence of malignancy was found. The CA19-9 level measured after the completion of the endoscopic treatment for common bile duct stones had increased to 6076 U/mL.

Surgical findings

**Figure 4:**
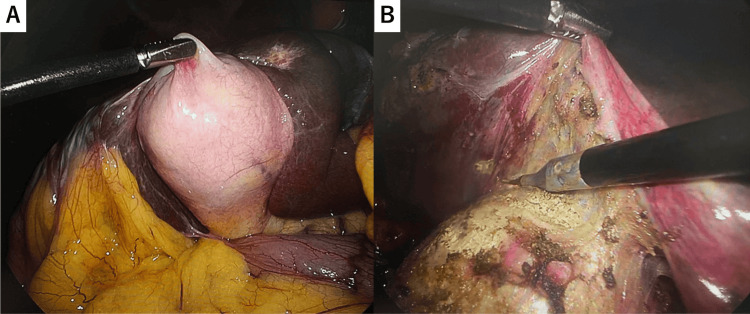
Laparoscopic cholecystectomy Laparoscopic cholecystectomy was performed. The gallbladder was hardened, but the serosa itself was not particularly stiff (A). Calcification did not extend to the gallbladder neck, and the cystic duct and cystic artery were clipped and divided in the usual manner before the gallbladder was dissected from the liver bed and removed (B)

Laparoscopic cholecystectomy was performed 21 days after admission. The gallbladder was hardened, but the serosa itself was not particularly stiff. Calcification did not extend to the gallbladder neck, and the cystic duct and cystic artery were clipped and divided in the usual manner before the gallbladder was dissected from the liver bed and removed (Figure 4).

Resected specimen and pathological findings

**Figure 5:**
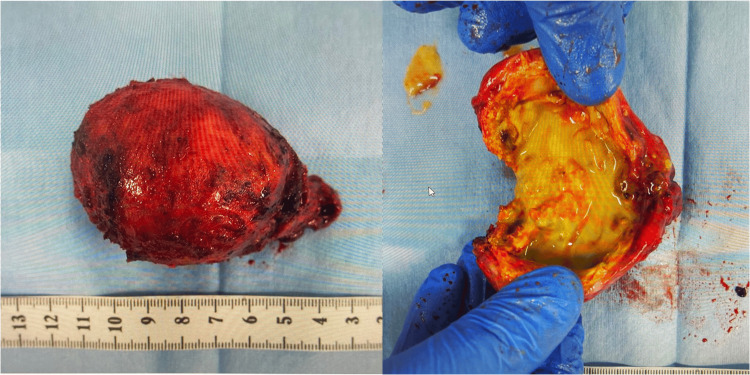
Resected gallbladder The resected gallbladder specimen showed no tumorous lesions, and yellowish-white fluid was present in the gallbladder

**Figure 6:**
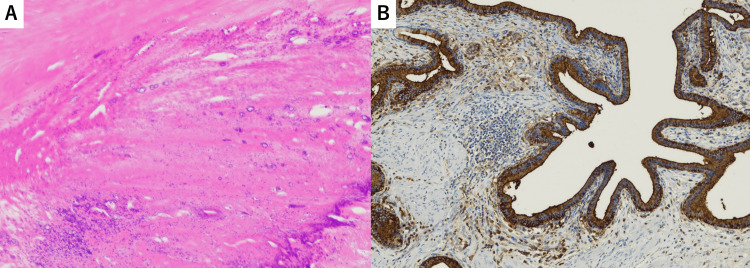
Histopathological findings Histopathological examination (microscopy) of the gallbladder surgical specimen marked fibrosis of the gallbladder, with calcification, ossification, hyalinization of the wall, and xanthogranulomatous inflammation (A: 20× H&E). Immunostaining for CA19-9 showed strong positivity in the gallbladder mucosal epithelium exhibiting xanthogranulomatous inflammation (B: 20× CA19-9 stain)

The resected gallbladder specimen showed no tumorous lesions, and yellowish-white fluid was present in the gallbladder (Figure 5). Histopathological examination revealed marked fibrosis of the gallbladder, with calcification, ossification, hyalinization of the wall, and xanthogranulomatous inflammation. Immunostaining for CA19-9 showed strong positivity in the gallbladder mucosal epithelium exhibiting xanthogranulomatous inflammation (Figure 6).

Postoperative course

The postoperative course was uneventful, and the patient was discharged on the seventh postoperative day. Blood tests performed in the outpatient setting showed that the CA19-9 level had returned to the normal range (17 U/mL).

## Discussion

Porcelain gallbladder is a rare condition characterized by an extensive calcification of the gallbladder wall, observed in 0.06%-0.8% of laparoscopic cholecystectomy cases [1,2]. It predominantly affects women, particularly those in their 60s, with a female-to-male ratio of 4-5:1 [3]. Chronic cholecystitis is thought to induce calcification, with the obstruction of the cystic duct or gallbladder neck, mechanical irritation, and persistent chronic inflammation leading to the repeated regeneration and necrosis of the gallbladder mucosa, which in turn progresses calcification and sclerosis [3].

Porcelain gallbladder is diagnosed based on imaging findings of calcification in the gallbladder wall. On plain abdominal radiography, it appears as eggshell-like or oval calcification in the right upper abdomen. CT scans clearly demonstrate the calcification of the gallbladder wall, which may appear as a donut-like or ringlike structure. Abdominal ultrasonography shows a curved echo with posterior acoustic shadowing [5,6].

The standard treatment for porcelain gallbladder is cholecystectomy. Previously, laparoscopic cholecystectomy for porcelain gallbladder was considered relatively contraindicated due to the potential difficulty in grasping or retracting the calcified wall, which could lead to complications [7]. However, current diagnostic imaging techniques, including preoperative ultrasound and CT, allow for the assessment of the extent of calcification, making laparoscopic surgery feasible [8]. In this case, laparoscopic cholecystectomy was performed without technical difficulties, as calcification did not extend to the cystic duct.

Traditionally, porcelain gallbladder has been associated with an increased risk of gallbladder cancer, with reported malignancy rates ranging from 12% to 33% [1,4]. Therefore, prophylactic cholecystectomy has been recommended for porcelain gallbladder due to the high risk of gallbladder cancer [9]. However, recent studies have reported that the incidence of gallbladder cancer is around 6%, which is not significantly higher compared to the 1% incidence in cases without calcification [10-12].

CA19-9 is a useful marker for diagnosing pancreatic and biliary cancers but can also reach abnormally high levels in benign biliary diseases. In particular, CA19-9 levels can exceed 1000 U/mL in cases of biliary obstruction or cholangitis [13]. Elevated CA19-9 levels in benign conditions are thought to result from leakage from the biliary epithelium due to obstruction or inflammation. In cases of obstructive jaundice, impaired bile excretion can lead to CA19-9 accumulation and abnormally high serum concentrations [14]. In this case, transient biliary obstruction due to common bile duct stones likely contributed to the sharp increase in CA19-9. Furthermore, the chronic inflammation associated with the porcelain gallbladder may have led to the continued leakage of CA19-9 from the gallbladder mucosa, resulting in the observed elevation.

Reports on abnormally elevated CA19-9 levels in porcelain gallbladder are scarce. In particular, cases of benign porcelain gallbladder with CA19-9 levels exceeding 1000 U/mL are extremely rare. Most existing reports have associated elevated CA19-9 levels with the presence of gallbladder cancer [15]. This case, involving a benign porcelain gallbladder with CA19-9 levels exceeding 6000 U/mL, is exceptionally rare.

Interestingly, elevated CA19-9 levels have also been reported in xanthogranulomatous cholecystitis, a benign inflammatory condition characterized by the accumulation of lipid-laden macrophages, fibrosis, and the chronic inflammation of the gallbladder wall. Xanthogranulomatous cholecystitis can lead to a significant elevation of CA19-9, sometimes mimicking malignancy due to the intense inflammatory response [16]. Similar to porcelain gallbladder, the chronic inflammatory process in xanthogranulomatous cholecystitis can cause the leakage of CA19-9 from the gallbladder epithelium, resulting in markedly high serum levels. This suggests that, in both conditions, the inflammatory process plays a crucial role in the abnormal elevation of CA19-9, underscoring the importance of careful differentiation between benign and malignant conditions.

In this case, the postoperative course was favorable, and CA19-9 levels normalized. Although elevated CA19-9 levels are an important indicator suggesting the possibility of malignancy, benign conditions can also present with abnormally high levels, requiring careful diagnosis. In particular, elevated CA19-9 levels in porcelain gallbladder are often due to biliary obstruction or chronic inflammation, and if malignancy is excluded by pathological diagnosis, the prognosis is considered favorable [14].

## Conclusions

This case reports a rare instance of a benign porcelain gallbladder with abnormally elevated CA19-9 levels, ultimately found to be nonmalignant. Although a significant elevation of CA19-9 is usually associated with malignant diseases such as biliary or pancreatic cancer, benign conditions can also present with elevated levels due to biliary obstruction or chronic inflammation. Particularly in cases like this one, where CA19-9 levels exceeded 6000 U/mL, careful differentiation between benign and malignant conditions is necessary. Furthermore, this report highlights the importance of considering xanthogranulomatous cholecystitis as another benign condition that may present with significantly elevated CA19-9, necessitating a thorough evaluation to avoid misdiagnosis.
